# Estimating Body Related Soft Biometric Traits in Video Frames

**DOI:** 10.1155/2014/460973

**Published:** 2014-07-09

**Authors:** Olasimbo Ayodeji Arigbabu, Sharifah Mumtazah Syed Ahmad, Wan Azizun Wan Adnan, Salman Yussof, Vahab Iranmanesh, Fahad Layth Malallah

**Affiliations:** ^1^Department of Computer and Communication Systems Engineering, Universiti Putra Malaysia (UPM), 43400 Serdang, Selangor, Malaysia; ^2^Department of Systems and Networking, Universiti Tenaga Nasional, Jalan IKRAM-Uniten, 43000 Kajang, Malaysia

## Abstract

Soft biometrics can be used as a prescreening filter, either by using single trait or by combining several traits to aid the performance of recognition systems in an unobtrusive way. In many practical visual surveillance scenarios, facial information becomes difficult to be effectively constructed due to several varying challenges. However, from distance the visual appearance of an object can be efficiently inferred, thereby providing the possibility of estimating body related information. This paper presents an approach for estimating body related soft biometrics; specifically we propose a new approach based on body measurement and artificial neural network for predicting body weight of subjects and incorporate the existing technique on single view metrology for height estimation in videos with low frame rate. Our evaluation on 1120 frame sets of 80 subjects from a newly compiled dataset shows that the mentioned soft biometric information of human subjects can be adequately predicted from set of frames.

## 1. Introduction

Many advances have been made in the domain of biometrics using physical or behavioral attributes like face, iris, fingerprint, hand geometry, voice, and signature for recognizing individuals [1, 2]. Traditional biometrics possesses high level of uniqueness, permanence, and distinctiveness. As a result, studies carried out on these traits have led to several significant and interesting findings. For instance, iris and fingerprint have been found out to be the most reliable biometric traits used for recognition. Likewise, face recognition is now a very popular and widely used mode of recognition across various fields [3]. In addition, many state-of-the-art algorithms have been proposed with optimum accuracy [4–6]. However, the level of intrusiveness, human compliance, computational cost, and time are amongst the disadvantages of these attributes.

Recently, soft biometrics has been introduced as a recognition technique that can make use of labels, measurements, and descriptions of individuals in surveillance or long distance videos for recognition in an unobtrusive and nonuser compliant way [7, 8]; although they cannot uniquely identify a particular individual due to lack of distinctiveness and permanence [9]. Advantages of soft biometrics include low computational cost and time. The data collection is user friendly and identification is enrolment free. More so, they can be used for filtering a large database, identification and reidentification of individuals, and the ability of being used at a distance in a less constrained environment. Utilizing a single soft biometric trait can be rather vague to an extent. But, in an event where traditional biometrics cannot be deployed due to constrain of distance between the subject and acquisition system, also, in a limited or group specific application whereby the number of users is relatively small and/or optimum accuracy is not generally required, combining a variety of soft biometric attributes can act as an intelligent identifier without the consent of the individuals. Therefore, they can be applied to limit the search for identity to a small category from a large pool of subjects, as such reducing the problem of distinctiveness and permanence, and at the same time providing detailed descriptions for recognition.

In previous literatures, soft biometrics has been mainly categorized into face-based, body-based, and accessory-based [10]. In the work carried out by Dantcheva et al. [11], the authors made use of several face related soft biometrics (like eye color, hair color, skin color, beard, and moustache) for face recognition. The traits were used to place subjects into several groups; then, the probability of collision between two randomly chosen authentication groups was used to measure the recognition rate of the system. Also, combination of face and soft biometric information (like skin and clothes color) were used to perform continuous authentication by [12]; since biometric systems only perform initial authentication at the beginning of a session, it can subject the system to possible impostor threat. As such, soft biometrics was used to occasionally update the identity of the users when they are not making any serious interaction with system. In addition, frontal to side face reidentification was carried out in [13], using both face- and accessory-based soft biometrics (like hair color, skin color, and clothes color).

While facial traits are more suited to fast recognition from close distance, body related attributes have been more adapted to surveillance environments. Denman et al. [14] proposed an approach for identification of individuals using size and clothes color across several camera views. The authors segmented the images into head, torso, and leg region for the estimation of height, relative size, and also color information. Velardo et al. [15] used estimated height, weight, and clothes color for identification across two camera views with protection of individuals' privacy. The previous works have merely utilized the body related soft biometric information for recognition, without actually considering the level of reliability of the prediction or estimation of the attributes, which can be important in matching the correct identity.

In this study, we focus on predicting two main body related soft biometrics traits, which are height and weight in an indoor environment. The possible application of the proposed method could be adapted to visual based patient caring and security guarding [16, 17]. The contribution in this paper is to evaluate the reliability of estimation of the attributes under changes in appearances and low video frame rate. We adopt the concept of single view metrology for height estimation and we propose a new computational approach to contactless weight estimation from video frames, whereby the subjects have varying appearances in an unconstrained indoor environment. Height and weight have been specifically focused on in this study because they are attributes that can be inferred from distance when facial information cannot be easily constructed. The rest of the paper is structured as follows.  Section 2 presents the experimental dataset and describes the technical details and strategies adopted for compiling the data. In Section 3, the materials and methods utilized for the proposed system are explained in detail.  Section 4 shows the experimental results achieved, and we summarize our work in Section 5.

## 2. Experimental Dataset

Our approach to this data collection is to compile soft biometric information in a free and naturalistic way in an unconstrained indoor environment using commercially available single view camera. Previous data used for soft biometrics research are usually annotated from gait dataset like CASIA [18] and video surveillance datasets like PETS 2006 [19] and VIPER [20], whereby subjects are tracked across multiple camera networks. Although the use of multiple cameras provides additional information about the scene and object being tracked, the practicality of using multiple acquisition systems for visual surveillance, especially in an indoor environment, incurs more computational cost and complexity. A single view camera system can be easily implemented and very affordable and the computational complexity involved is much minimized, whilst providing adequate information for recognition.

Moreover, there is currently no publicly available dataset to specifically evaluate the performance of full body measured information such as height and body weight model estimated from video.

### 2.1. Data Acquisition

**Figure 1:**
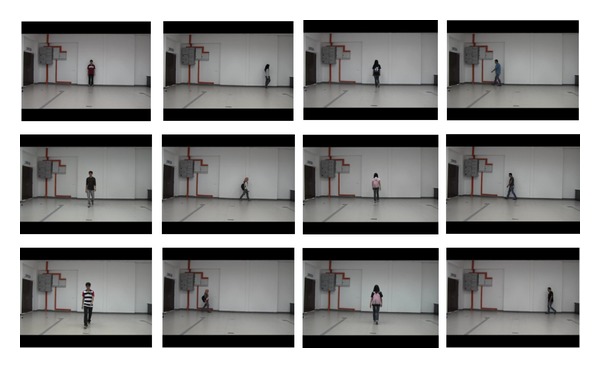
Sample images of four subjects appearing at three different times, covering four different directions, with variation in appearances.

The dataset, UPM SOFTBIO, was captured and recorded in the Computer and Embedded Systems Laboratory, Universiti Putra Malaysia. The dataset was compiled between May and June 2013, in an indoor environment under uncontrolled illumination. It involved 101 subjects, who willingly volunteered to participate. The entire process was divided into 3 sessions in order to accommodate more intra- and intersession variability, as shown in Figure 1.

In the first session, we assigned a user ID and registered each subject with their metadata like age and gender and manually measured each subject's respective height and weight. After registration was completed, the only instruction given to the subjects was to walk towards and across the camera view at their individual normal stride rate. In the second and third sessions, the subjects were captured in their changed appearances, for instance, carrying backpack and changed clothes, shoes, and hair style.

There was an overall 15-day period between each subsequent session. All the 101 subjects successfully completed the first session, while 80 subjects appeared in two sessions, and 70 subjects were able to participate in the third session. The camera is a single view camera, recording at 50 frame rate per second, with resolution of 1440 × 1080. The height of the camera from ground plane is 120 cm, positioned at a distance of 700 cm to the subjects. The walk across the camera was performed to cover a distance of 600 cm, which depicts full profile of left and right side pose. The walk towards the camera covered 400 cm, representing full profile of frontal and back views and we allowed an extra 100 cm for subjects to be able to make a turn to another direction. Some landmark points were placed on the floor of the camera field of view to indicate where the subjects should start and end the walk.

### 2.2. Technical Details

The dataset has been annotated and organized based on pose, appearance, and time variability. The first set is composed of frame sequence and video recordings from the first session recordings. The second set composing different appearances from the second session is divided into two groups; articulation with backpack and articulation without backpack. Also, the third set, containing another different appearance from the third session, is divided in the same way as the second set. An empty background was recorded for each participant at the end of the walk.

The time required for a participant to complete the data collection task is ~4 mins, starting from the manual collection of the metadata like height, weight, gender, age, and ethnicity to the completion of the walk. The recording of the walk of each participant elapsed between 35 sec~1 min 10 sec, depending on the stride rate. The minimum number of frames is ~1750, while the maximum number of frames is ~3500. The camera has a storage capacity of 64 GB; as such the transfer of data was performed at the completion of each session. The frames and metadata for each participant have been extracted and stored on the desktop and external hard drive with a folder for each subject, which we annotated with user numbers.

### 2.3. Demographic Details

**Figure 2:**
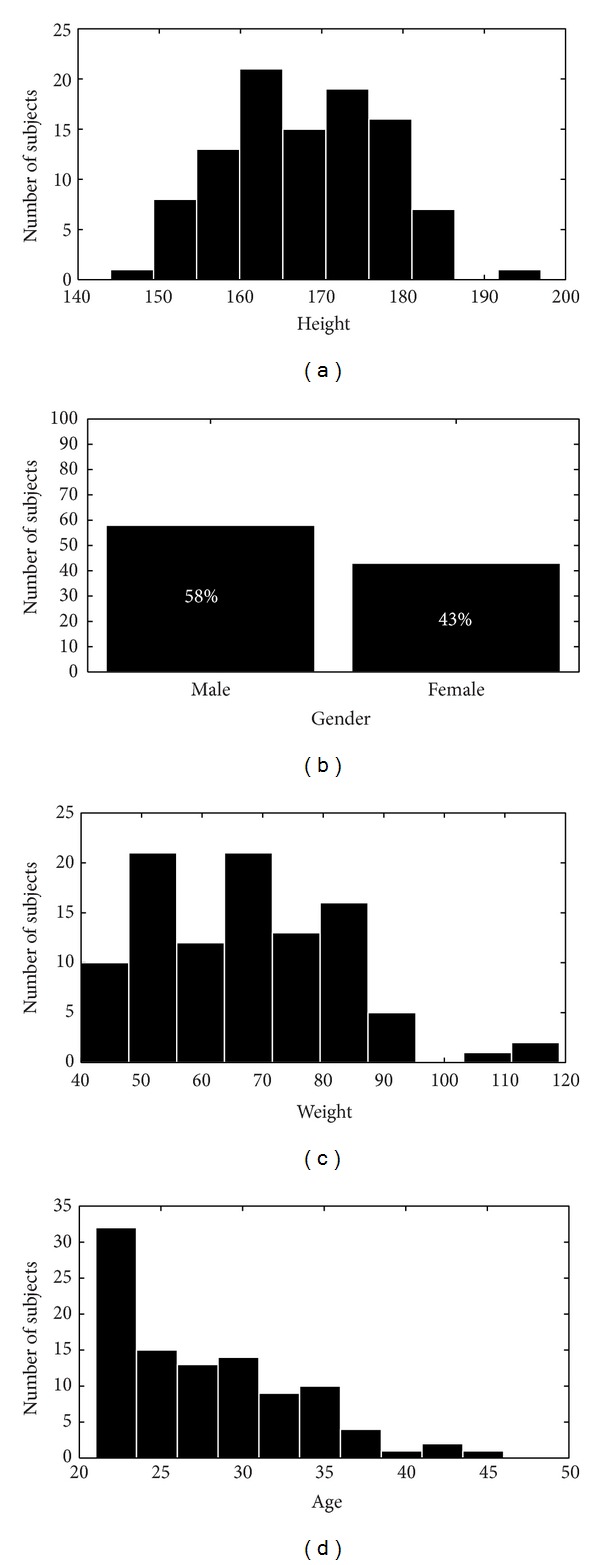
Plot of metadata of subjects according to height, gender, weight, and age distribution.

Demographic distribution is a very important tool in soft biometrics research. The 101 subjects that participated in the data compilation were from different ethnicities, gender, and age group. 51% were from South Asia (29% Chinese, 22% Malay), 28% were from the Middle East, 16% were from Central Asia, and 6% were Africans. 58% were male and 43% female and 62% were between 20 and 30 years, while 39% were between 31–45 years. The height range was between 144 and 197 cm and the weight was between 40 and 119 kg. The distribution of the subjects is depicted in Figure 2.

## 3. Materials and Methods

The techniques we propose are to compute the weight of moving subjects using features from the body and feed forward neural network (FFNN), and also to adapt the concept of single view metrology to low frame rate video for estimating upright human height.

### 3.1. Object Extraction

**Figure 3:**
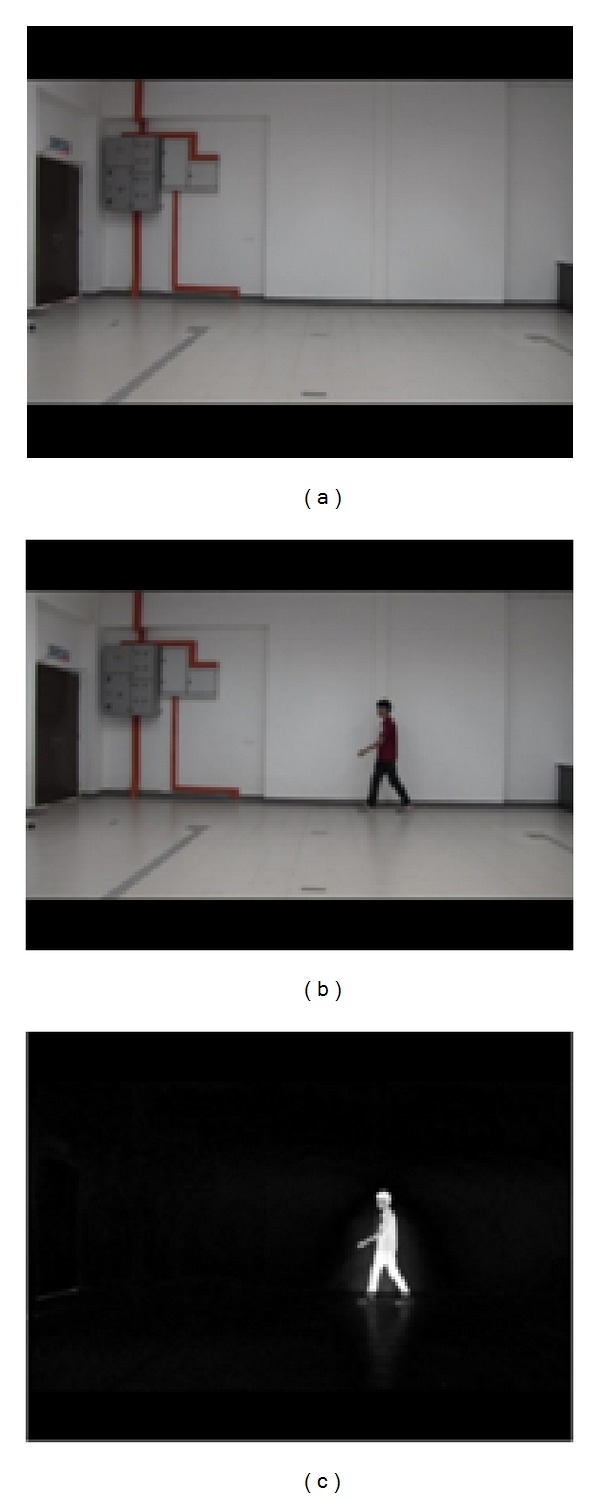
Process of object extraction. (a) Empty background frame. (b) New frame with walking subject. (c) Difference image.

Extraction of the object as silhouette from the background is a very important step for the proposed system. In this work, the steps for the object extraction include background subtraction and shadow removal from the extracted object. Consider an input frame of an empty background, bg, and a current frame, curr, containing a moving subject, where all the pixels are within the RGB color space. The difference, *D*, between the two frames is computed using

(1) $$\begin{array}{c} D\left(x,y,i\right)=\sqrt{\text{sum}{\left[\left(b\left(\text{bg}\left(x,y,i\right)\right)\right)-\left(b\left(\text{curr}\left(x,y,i\right)\right)\right)\right]}^{2}\;}, \end{array}$$

where *b* is the *R*, *G*, *B* band of each frame and *D* is a difference image resulting from the subtraction of the two frames, as shown in Figure 3.

Then, to determine the foreground *F*_*M*_, Otsu thresholding [21] technique is used to automatically define a threshold *T*_thres_. But, in this experiment, we introduced a threshold suppression parameter, *c*, in (2), which is a value greater than 1:

(2) $$\begin{array}{c} F_{M}\left(x,y,i\right)=\{\begin{array}{cc} 1, & \text{if}\;D\left(x,y,i\right)>\frac{T_{\text{thres}}}{c}\\ 0, & \text{otherwise}. \end{array} \end{array}$$

**Figure 4:**
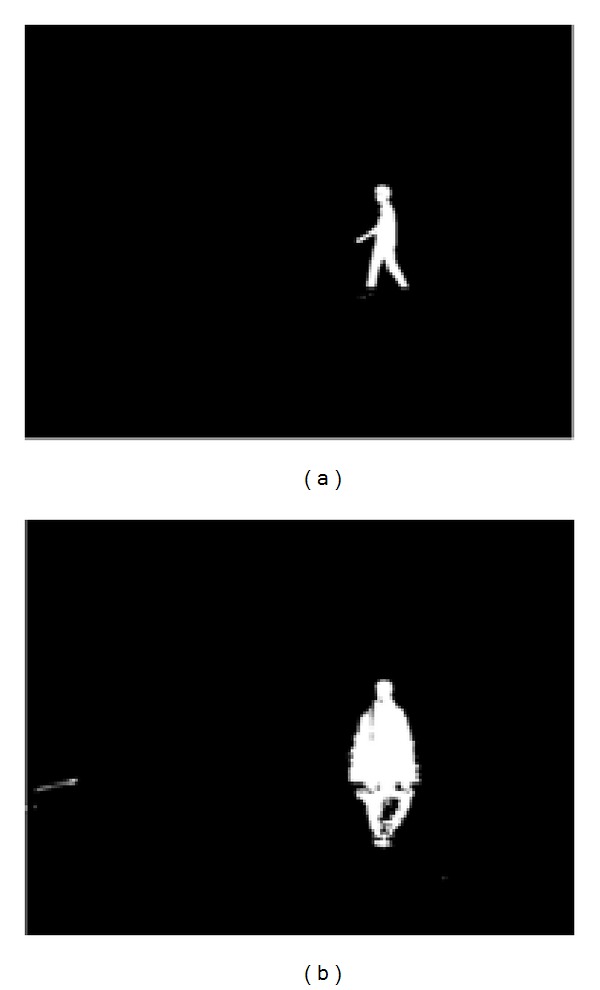
Threshold suppression. (a) Initial segmentation using Otsu threshold. (b) Segmentation by adding parameter *c*.

The reason for introducing parameter, *c*, is to enhance the contrast of the foreground image. Furthermore, the determined threshold *T*_thres_ is observed to be too strict, as it segments the leg region of most subjects as part of background; an example is shown in Figure 4(a). This is because Otsu technique mainly depends on bimodal distribution of histograms of the two classes. Thus, effect of varying illumination can significantly influence the computation of class variance.

**Figure 5:**
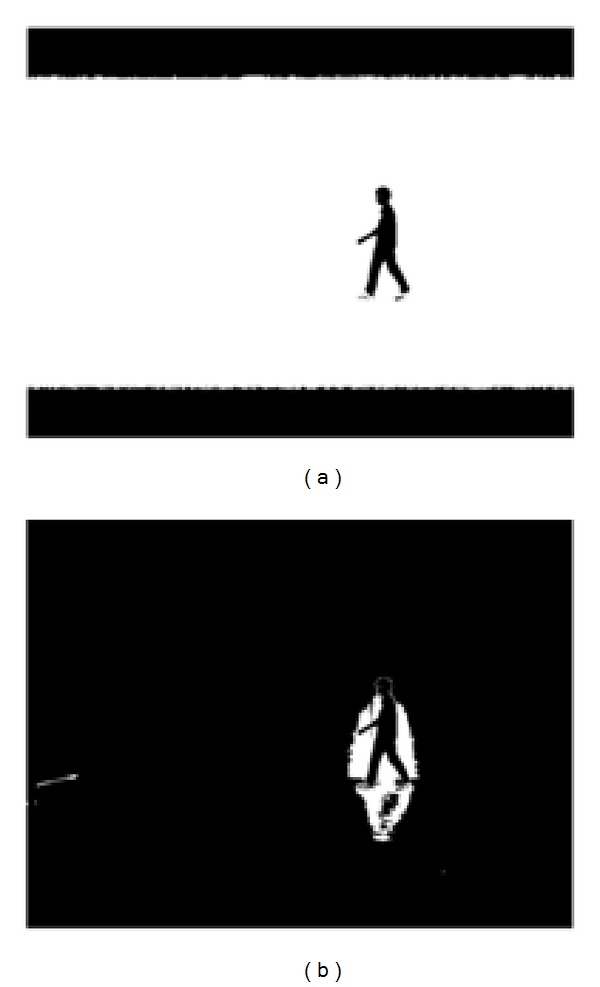
Highlighting brightness difference around moving foreground pixels.

Although improving the contrast consequently results in considering incidental pixels, due to shadow cast, as part of the foreground image, in order to remove the shadows that are extracted with the object, a color and brightness difference, *B*_*D*_, is computed as [22]

(3) $$\begin{array}{c} B_{D}=\left|\log\left(\frac{I_{b}}{I_{f}}\right)\right|+D_{\text{FB}}, \end{array}$$

where *D*_FB_ is the difference between the new frame pixels and the background frame in the normalized GB space, while *I*_*b*_ is background brightness, and *I*_*f*_ is new frame brightness, in RGB space. If *B*_*D*_ < *T*, which is empirically estimated, it is regarded as lighting changes, so the pixels are removed. The output is a brightness difference image, shown in Figure 5(a).

But to highlight the brightness difference around the moving object, as shown in Figure 5(b), we used a simple logical AND operator, in the following expression:

(4) $$\begin{array}{c} \text{Shadow}=F_{M\;}\text{AND}\;B_{D}. \end{array}$$

Finally, to extract the well enhanced foreground image, *F*_img_, the following expression is utilized:

(5) $$\begin{array}{c} F_{\text{img}}=F_{M\;}\text{AND}\left(1-\text{shadows}\right). \end{array}$$

**Figure 6:**
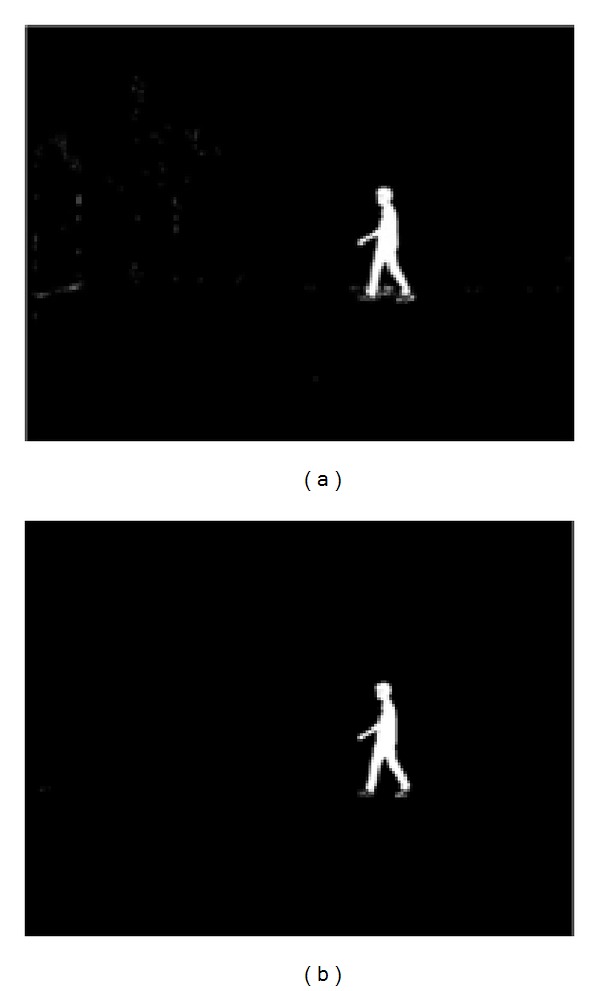
Resultant foreground image. (a) Foreground with random noise (b) Postprocessed foreground image.

The resultant is a clear foreground image, *F*_img_, with some random noises, considered as small regions that are post processed by applying morphological operations, as shown in Figure 6. The described technique performs effectively well for our task since the experiment is limited to indoor environment. Though, we note that, in more challenging outdoor scene, object detectors based on more robust local features can be deployed [23, 24].

### 3.2. Height Estimation

Several methods have been proposed for estimation of human height. There are two main techniques, one of which includes the use of intrinsic parameters of the camera retrieved from camera calibration [25, 26]. The other technique usually referred to as uncalibrated technique includes the use of information from the scene as extrinsic parameters of the camera. The aim in this method is to accurately locate the unknown camera centre or position in the image view with respect to a known reference frame. The most popular one based on 3D affine measurements was presented by Criminisi et al. [27]. Our estimation is mainly based on the technique proposed by Criminisi et al. (see [27]), but quite notable from their work is that a single image has been used for height estimation of stationary objects. This experiment incorporates the technique for estimating height of moving objects in frames of video that possess extremely low frame rate and we used only the height of the acquisition camera as a reference height. Also, the object extraction and vanishing point estimation are performed automatically.

In the concept of single view metrology, if we consider the top and bottom points *x*_*t*_ and *x*_*b*_ of an object in 3D real world view and, also, consider another point *y* in the same field of view, the distance *d* between *y* and *x*_*b*_ represents the height of an object. Distance, *d*, in this case is regarded as the reference height in real world measurement. Briefly note here that points *x*_*t*_ and *x*_*b*_ are on different planes. Hence, a line projection from the two planes, which tend to infinity, results in another point *v*, referred to as the vanishing point. As a result, *x*_*t*_, *x*_*b*_, *y*, and *v* mark a set of collinear points in the world coordinate. Therefore, a simple cross-ratio between the points can be used to obtain the objects height *H* in real world view. However, in the 2D image view, the projection of points *x*_*t*_, *x*_*b*_, and *v* denote image points *X*_*T*_, *X*_*B*_, and *V*. Hence, the main problem is estimating *V*, which is also the camera position in the image. If *V* is known, then the object's height *O*_*H*_ can be estimated, by calculating the proportion of the camera with respect to points *X*_*T*_, *X*_*B*_, at the current position of the object in the image view. Once we are able to represent the camera position in image, then, we computed height as follows:

(6) $$\begin{array}{c} O_{H}=\left(\frac{H_{C}}{V}\right)*\left(X_{T}-X_{B}\right), \end{array}$$

where *X*_*T*_ is top of the ROI boundary, *X*_*B*_ is bottom of the ROI boundary, *V* = *X*_*B*_ − *Y* is coordinate of the horizontal vanishing line, and *H*_*c*_ is height of camera (reference height).

### 3.3. Weight Estimation

Human weight is another body based attribute which represents the stature along with height. Motivated by recent advances in computer based image analysis, whereby there is a common interest in predicting human measures directly from image, conventional methods make use of weighing scale for measuring body weight. This method is not useful in many conditions whereby the estimate of human weight is considered vital information, for instance, situations whereby an offender is described by a human observer based on the estimate of the body weight [28] or in visual surveillance reidentification [15]. Therefore, to predict the body weight, the only measures that can be performed are restricted to the image frames acquired by the camera. It is important to emphasise that precise measures can be significantly influenced by noise, since the features are extracted from image.

Nevertheless, we gain an insight into this problem using computational intelligence methods. To the best of our knowledge, related works on weight estimation from image are very limited. Previously, Velardo and Dugelay [29] presented a method for weight estimation, by mapping manually extracted anthropometric measures of the whole human body to their respective weight using linear regression model for static subjects from frontal-to-side view. More recently, Labati et al. [30] proposed a computational intelligence approach to weight estimation, whereby the length, area, and volume measurements were automatically extracted across eight segments of the body relative to height at each segment as features. Neural network was used for mapping between measurements and weight of subjects. The estimation was performed with two calibrated cameras for 20 subjects. Most of the previous experimentations were carried out on limited datasets captured at the same time (day). Hence, the robustness of their techniques against appearance changes is not proven. The proposed method in this paper takes into consideration the effect of appearance changes as well as large fluctuation in strides of subject as a result of low frame rate.

**Figure 7:**
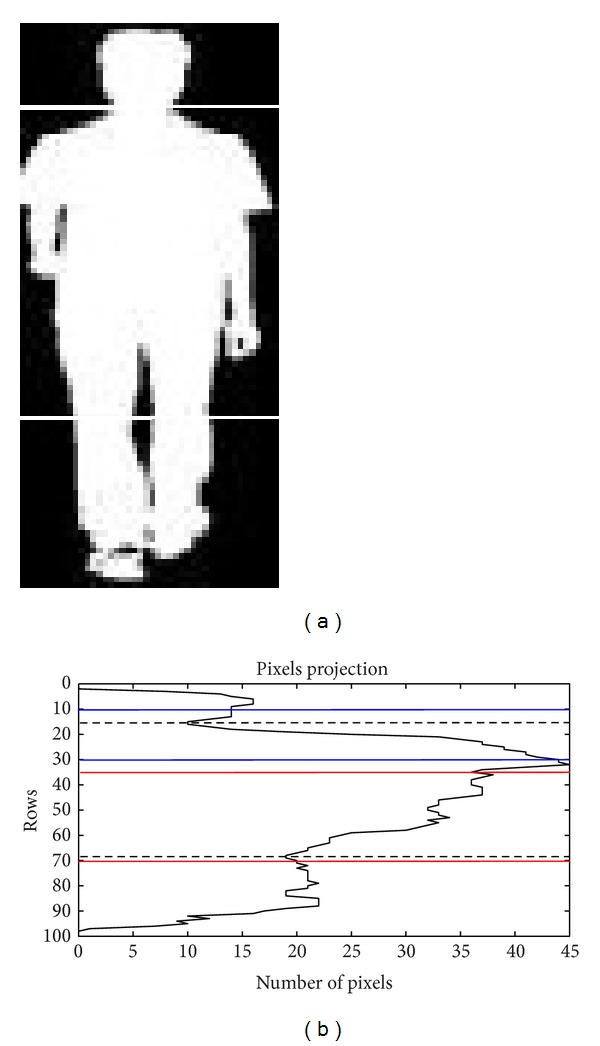
The region segmentation of an object. (a) represents the head torso and leg region and (b) depicts the horizontal projection, with the minimum points of head and torso highlighted with the dotted lines.

We take advantage of the background subtraction by segmenting the foreground image into head, torso, and leg regions [14], as shown in Figure 7. In order to determine the regions of segmentation, we first calculate the horizontal projection of all foreground pixels of the object using (4). Consider

(7) $$\begin{array}{c} P_{H}\left(j\right)=\overset{M}{\underset{i=0}{\sum }}B\left(i,j\right), \end{array}$$

where *P*_*H*_ is the horizontal projection and *B* is the binary image. Then, the regions of segmentation are automatically located as the minimum points using the following equations [14]:

(8) $$\begin{array}{c} H_{\text{region}}=\;\underset{i=0.1*I}{\overset{0.3*I}{\text{argmin}}}\left(P_{H}\left(j\right)\right)\\ T_{\text{region}}=\;\underset{i=0.35*I}{\overset{0.7*I}{\text{argmin}}}\left(P_{H}\left(j\right)\right), \end{array}$$

where *H*_region_ is the head region, which is located between the first 10 and 30 percent of *P*_*H*_, while *T*_region_ represents the torso region, located between 35 and 70 percent of *P*_*H*_, as illustrated in Figure 7.

From the three regions, twelve additional features are computed, in addition to the result from object height estimation, *O*_*H*_. For each image *I* of a subject, the pixel densities *H*_feat_, *T*_feat_, and *L*_feat_ of head, torso, and leg region are calculated using the following:

(9) $$\begin{array}{c} H_{\text{feat}}=\overset{n_{i}}{\underset{n=1}{\sum }}\frac{\left(I_{H}\right)}{L_{HP}}\\ T_{\text{feat}}=\overset{n_{i}}{\underset{n=1}{\sum }}\frac{\left(I_{T}\right)}{L_{TP}}\\ L_{\text{feat}}=\overset{n_{i}}{\underset{n=1}{\sum }}\frac{\left(I_{L}\right)}{L_{LP}}, \end{array}$$

where *L* is the length of the segmented region in the image. Further, the size of the object, *W*_feat_, is computed by dividing the area of the silhouette by difference of the head and bottom point in pixel:

(10) $$\begin{array}{c} W_{\text{feat}}=\frac{\text{Area}\left(I\right)}{\left(X_{T}-X_{B}\right)}. \end{array}$$

The weighted ratio of the pixels of the head, torso, and leg region, *R*_*H*_, *R*_*T*_, and *R*_*L*_, is calculated by giving more significance to the numerator:

(11) $$\begin{array}{c} R_{H}=\frac{{\left(H_{\text{feat}}\right)}^{2}}{T_{\text{feat}}+L_{\text{feat}}}\\ R_{T}=\frac{{\left(T_{\text{feat}}\right)}^{2}}{H_{\text{feat}}+L_{\text{feat}}}\\ R_{L}=\frac{{\left(L_{\text{feat}}\right)}^{2}}{H_{\text{feat}}+T_{\text{feat}}}. \end{array}$$

Also, the lengths of head, torso, and leg regions and objects height in centimeters (cm) are computed by using the following expressions:

(12) $$\begin{array}{c} L_{L}=\left(\frac{H_{C}}{V}\right)\times \left(L_{LP}\right)\\ L_{T}=\left(\frac{H_{C}}{V}\right)\times \left(L_{TP}+L_{LP}\right)\\ L_{H}=\left(\frac{H_{C}}{V}\right)\times \left(\left(X_{T}-X_{B}\right)-\left(L_{TP}+L_{LP}\right)\right). \end{array}$$

**Figure 8:**
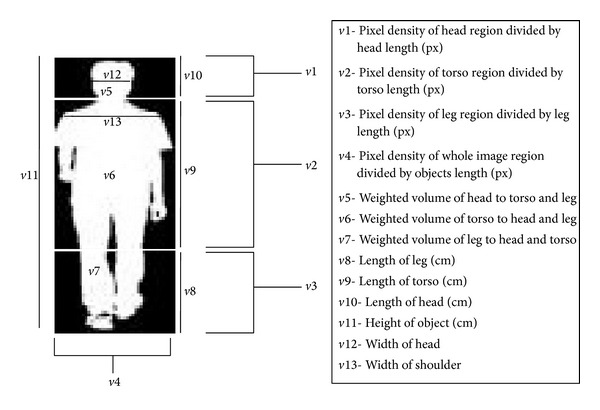
The feature set for weight computation.

Finally, the width of the head *W*_*H*_ and width of shoulder *W*_*S*_ of the subjects are calculated by using region label techniques, whereby the pixels which belong to white are searched based on the connected components and the maxima of summations of all connected components are selected. For head, the search is limited to the midlevel of the head region, while, for the shoulder region, the search is restricted to the first 10 percent of the torso binary image, as shown in Figure 8.

As a result, 13 feature sets [*v*1 ⋯ *v*13] are considered for weight estimation. Furthermore, we exploited a computational intelligence method by passing the extracted feature vector to FFNN, to compute the weight of each subject represented in kg. Before modeling FFNN, the technique described in [31] is adopted for normalizing the features between the range of 0 and 1.

## 4. Experimental Results

**Figure 9:**
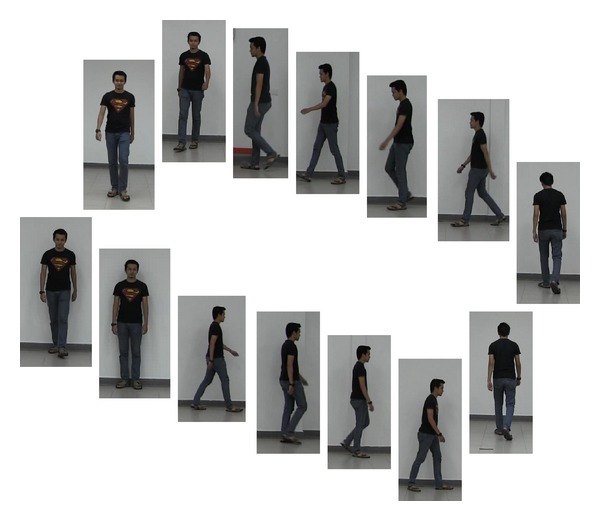
Fourteen frame sets of a subject selected at 1 fps showing four different poses.

We implemented the proposed system using 1120 video frames of 80 subjects from our newly compiled dataset, UPM SOFTBIO, with each subject walking across and towards the camera representing four different poses. For this experiment, the frames are selected at 1 frame per second (fps) representing as low frame rate as possible, in order to provide a large fluctuation in the strides of the subject as shown in Figure 9.

### 4.1. Height Estimation

**Figure 10:**
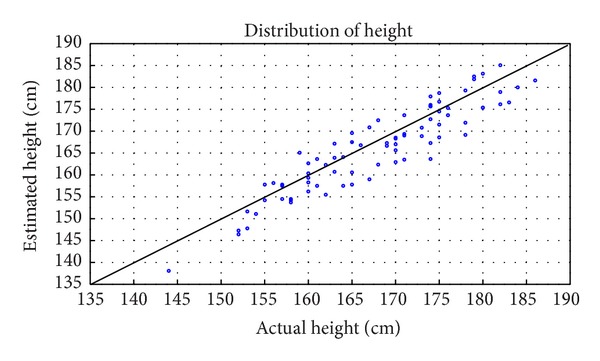
Plot of actual against estimated height by incorporating single view metrology to low video frame rate (1 fps).

In order to evaluate the height estimation using single view metrology, the object extraction is utilized to detect the ROI to retrieve the top and bottom coordinates of the subjects, before computing the height using (3). Based on that, the model attained a mean absolute error (MAE) of 1.57 cm and standard deviation of 3.6 cm for the 80 subjects. This denotes that even though there is large fluctuation in the strides of the subjects due to low frame rate, the errors attained in a particular frame can be well compensated by the accurate prediction in the subsequent frames. Besides, the result is very comparable to the related works on height estimation in video with single view camera, by considering the error attained with respect to the frame rate of the video as shown in Table 1. In addition, the scatter plot of distribution of estimated height against actual height for all 80 subjects in the database is represented in Figure 10 and the results achieved for the first 20 subjects are presented in Table 2.

### 4.2. Weight Estimation

For body weight estimation, the data from session 1 is used for training FFNN, with single hidden layer. The Levenberg-Marquardt training function is used for back propagation. The hidden layer uses the logistic sigmoid (logsig) activation function, while linear (purelin) activation function is utilized in the output layer. During training, the data is randomly divided into 70 and 30 percent as training and validation set, respectively. The criteria for stopping the training, in order to avoid over fitting, are based on any of the following three conditions accordingly:

- in every iteration, if the optimizer is able to reach convergence, once validation is performed, the training should be stopped;
- if the validation error starts to increase for six consecutive attempts, the training should be stopped;
- the training should be stopped if the maximum number of epoch, 100 is attained.

Then, after training is completed, we tested the ability of neural network to learn and adapt from recognized patterns, even in the presence of noise as a result of changes in appearance of the subjects, using a different data acquired in session 2, with a difference of 15 days to the training data.

**Figure 11:**
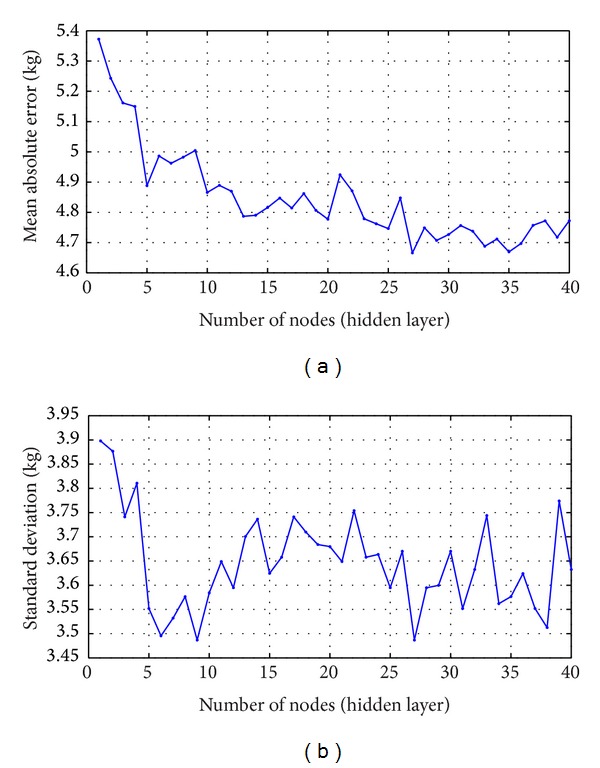
Performance of different nodes. (a) Mean absolute error and (b) standard deviation.

It is more important to note that, in order to determine the best parameter for the body weight estimation using FFNN, an initial experimentation is performed using different number of nodes from 1 to 40, whereby the experiment on each node is run 10 times, with different seeds and the results of the performance of the nodes are plotted in Figure 11. We present our results in terms of mean absolute error (MAE) and standard deviation. The final result for each node is the average of the results from the 10 runs. The best result for weight estimation for all 80 subjects in the database is MAE of 4.66 kg and standard deviation of 3.48 kg. The result is attained with single hidden layer neural network of 27 nodes.

**Figure 12:**
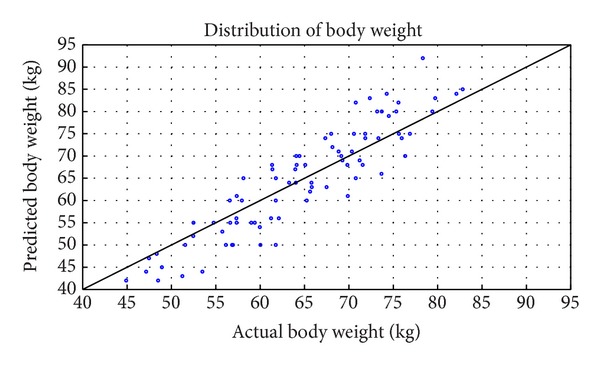
Plot of actual against estimated body weight attained at 27 nodes.

The scatter plot of distribution of predicted against actual body weight for the whole 80 subjects in the database is shown in Figure 12. Also, in Table 2, is the result for the first 20 subjects in the database.

Velardo and Dugelay [29] have presented a baseline body weight estimation model and confirmed that their system could predict the body weight of 20 static subjects with an error of ±5 to the real weight of the subjects from image, while Labati et al. [30] performed weight estimation of 25 subjects with two calibrated cameras. The authors reported their result with* mean error*, as they attained a mean error of 0.07 kg and standard deviation of 2.3 kg. However, the dataset used for the experiments is not publicly available.

Moreover, since the proposed method in this paper is based on single view camera, therefore, to offer a fair comparison of our approach, the proposed method is benchmarked against the baseline model of Velardo and Dugelay [29], by implementing their feature extraction technique and model on our dataset. The result is highlighted in Table 3. With regard to that, the proposed technique is more robust and outperformed the technique presented in [29] by a significant factor in terms of fluctuation in strides of the walking subjects and changes in clothes appearance. The mean absolute error (MAE) and standard deviation of the two models are highlighted in Table 3.

Some important observations that can be inferred which indicate the advantage of the proposed method over the model in [29] are pointed out as follows.

- The proposed technique is implemented on dataset of walking subjects, whereas in [29] frontal and side view image pair of static subjects were used for deriving the body weight model. Therefore, the robustness of their work on data with fluctuations in strides of the subjects is not analyzed, which is evident in the result attained when we implemented the technique on UPM SOFTBIO, as shown in Table 3. In addition, our proposed method performs reliably despite changes in appearance of the subjects.
- The model in [29] requires a very precise and accurate localization of feature points due to the need to measure the geometric length or width between the interest points. Despite the fact that the geometric measures are performed manually, we discovered that an accurate and precise localization can be significantly affected by large clothes of the subjects and self-occlusion caused by arm's swing while the subjects are in motion. But the proposed method in this paper does not necessitate a precise localization of feature points, since it utilizes an automatic procedure of coarse segmentation of the subjects into 3 main body regions.

In order to offer completeness of the proposed technique and ensure generalization of our analysis, an additional experimentation is performed using the optimal number of nodes, 27, for the hidden layer of FFNN to evaluate whether the model can predict the body weight of an unknown subject. For this purpose, session 1 data is randomly divided into equal halves, whereby the first 50% (560 frames) is used for training and the remaining (560 frames) is used for testing. The process is repeated 100 times with different partitions in each run and the final result is the average of 100 runs, presented in Table 4.

Finally, a further experiment is carried out by combining height and weight for a simple human identification at a distance. We used the matcher described in [36] for similarity matching of session 1 and session 2 data. In fact, the results are promising; even though the attributes are not predicted with optimum accuracy, the combination of the two attributes could attain a top rank identification of 51% and a rank 5 identification rate of 93%, while, at rank 10, the identification rate is 100%. Definitely, a reliable identification system cannot be modeled using height and weight alone, but the two attributes could serve in improving the performance of other biometrics such as face and gait recognition, either as a prescreening filter or by score fusion.

## 5. Conclusions

We implemented an approach for weight computation and height estimation from video frames in an unobtrusive manner. We experimented on 1120 frames of 80 subjects in a new dataset, UPM SOFTBIO, compiled in our laboratory which contains 101 subjects, walking in an indoor environment, under uncontrolled lightning conditions describing four different directions and varying appearances. The height estimation was based on existing techniques, but we incorporated the technique into video recording with 1 fps, thereby showing significant fluctuation in the walk of the subjects. Moreover, we utilize the camera's real height as the reference height and automatically located the camera position as the distance between the horizontal vanishing line and the feet of the object at any position in the field of view. The result indicates that, under varying strides as a result of low frame rate, the errors attained in a particular frame can be well compensated by the accurate prediction other frames.

For weight computation, several literatures on soft biometrics have suggested the possibility of using body weight as additional biometric information. However, only few literatures can be referred to which have actually gained an insight into this possibility using an image. Therefore, this paper demonstrated a technique for weight computation. We extracted features from the body segments of each individual after silhouette segmentation into head, torso, and leg regions and then used neural network to estimate their respective weights. It is worth noting that even though the result shows the ability of neural network to adapt to significant changes in objects' appearance, based on the MAE of 4.66 kg, we attribute the huge error attained to the effect of clotheses of the subjects. For instance, a subject, whose original body weight is 55 kg could be predicted as 59.66 kg, due to the change of clothes of the subject. Furthermore, the model could predict the body weight of an unknown individual with a MAE of 6.39 kg. This basically shows that the model performs well to a reasonable extent when only single image is available. The limitation of our work is that the experiment has been carried out only in indoor environment, although our target application is directed towards indoor visual surveillance. In future work, we will be incorporating more feature sets to the model and also compile additional datasets in outdoor environments to implement the proposed method.

## Figures and Tables

**Table 1:** Comparison of results of height estimation in video.

Technique	Frame rate (fps)	Error (cm)	Standard deviation (cm)
BenAbdelkader [32]	20	—	3.5
Nguyen and Hartley [33]	—	1.89	—
Jeges et al. [34]	—	—	4.3
Hansen et al. [35]	15∗∗	3.4	—
Our method	1	1.57	3.6

**Frame rate is presented in Hertz (Hz).

**Table 2:** Results of height and weight computation of the first 20 subjects in the database.

ID	Real height	Mean absolute error	Standard deviation	Real weight	Mean absolute error	Standard deviation
1	171	2	0	60	2.26	5.13
2	160	3.79	3.49	45	6.64	5.76
3	152	2.71	2.73	53	3.53	5.88
4	157	2.5	3.98	56	4.57	5.68
5	154	2.93	2.79	48	0.96	2.27
6	158	0.29	2.79	65	2.5	2.83
7	175	0.5	2.74	54	4.35	5.22
8	169	2.36	4.01	56	2.97	4.56
9	159	0.07	4.08	60	4.48	3.63
10	174	3.93	3.22	55	5.62	3.36
11	162	1.5	2.07	67	5.33	3.96
12	152	0.57	2.28	47	1.62	1.88
13	165	1.43	3.01	60	1.82	2.16
14	168	1.64	3.95	55	2.84	2.6
15	175	0.43	2.9	63	6.2	2.68
16	180	1.64	4.38	74	6.38	2.84
17	182	3.07	3.63	83	2.79	3.95
18	157	0.79	2.64	55	5.66	3.76
19	163	2.29	2.55	75	8.65	2.96
20	163	0.93	2.06	55	2.39	2.64

**Table 3:** Comparison of weight estimation with baseline model.

Technique	Feature extraction	Prediction model	MAE (kg)	Standard deviation (kg)
Velardo and Dugelay [29]	7 body measures	Linear regression	7.21	6.24
Proposed method	13 body measures	FFNN	4.66	3.48

**Table 4:** Results of weight estimation for unknown subjects.

Number of nodes	Training sample	Testing sample	MAE (kg)	Standard deviation (kg)
27	560 frames	560 frames	6.39	5.1
